# Temporal cross-correlations between air pollutants and outpatient visits for respiratory and circulatory system diseases in Fuzhou, China

**DOI:** 10.1186/s12889-020-08915-y

**Published:** 2020-07-20

**Authors:** Yu Jiang, Jiedong Chen, Chuancheng Wu, Xin Lin, Quan Zhou, Shumi Ji, Shuangfeng Yang, Xiaoyang Zhang, Baoying Liu

**Affiliations:** 1 Department of Preventive Medicine, Fujian Provincial Key Laboratory of Environment Factors and Cancer, Key Laboratory of Environment and Health, School of Public Health, Fujian Medical University, Fuzhou, China; 2 Fuzhou Center for Disease Control and Prevention, Fuzhou, China

**Keywords:** Air pollution, Coastal area, Time-series analysis, Season

## Abstract

**Background:**

Previous studies have suggested that there is an association between air pollutants and circulatory and respiratory diseases; however, relatively few have analyzed the association between air pollutants and outpatient visits based on the mortality, hospitalization rates, etc., especially in areas with relatively good air quality. Therefore, we conducted this study to research the association between air pollutants and outpatient visits in Fuzhou, China.

**Methods:**

We used a generalized linear Poisson model to study the association between air pollution and outpatient visits for respiratory and circulatory diseases from 2016 to 2018 in Fuzhou, China.

**Results:**

In the single pollutant model, nitrogen dioxide (NO_2_) had a significant effect. For lag day 0 to lag day 5, the effect decreased with every 10 μg/L increase in NO_2_. The daily maximum 8-h mean ozone (O_3_-8h) and upper respiratory outpatient visits were positively associated during the cold period [lag2, excess risk (ER) (95% confidence interval (CI)): 1.68% (0.44–2.94%)], while O_3_-8h and respiratory disease were positively associated during the warm period [lag5, ER (95% CI): 1.10% (0.11–2.10%) and lag4, ER (95% CI): 1.02% (0.032–2.02%)]. Similarly, particulate matter (PM) with an average aerodynamic diameter of less than 10 μm (PM_10_) and lower respiratory diseases were positively associated during the warm period [lag0, ER (95% CI): 1.68% (0.44–2.94%)]. When the concentration of O_3_-8h was higher than 100 μg/L, there was a positive effect on circulatory [lag5, ER (95% CI): 2.83% (0.65–5.06%)], respiratory [lag5, ER (95% CI): 2.47% (0.85–4.11%)] and upper respiratory [lag5, ER (95% CI): 3.06% (1.38–4.77%)] outpatient visits. The variation in O_3_-8h changed slightly when we adjusted for other air pollutants, and after adjusting for O_3_-8h, the ERs of the other air pollutants changed slightly. After adjusting for PM with an average aerodynamic diameter of less than 2.5 μm (PM_2.5_), the ERs of the other air pollutants increased, and after adjusting for NO_2_, the ER of PM decreased.

**Conclusion:**

Exposure to ambient NO_2_, O_3_, PM_2.5_ and PM_10_ was associated with an increase in respiratory and circulatory system-related outpatient visits in Fuzhou, China.

## Background

Ambient air pollution is on the rise, with the most marked increases in rapidly developing and industrializing low-income and middle-income countries [1]. As the largest developing country, China has developed increasingly serious air quality problems, and air pollution issues are increasingly prominent. However, few Chinese cities have established citywide morbidity reporting systems, and there are few studies of China’s coastal area [2, 3]. Data revealing the association between air pollution and human health are limited in China, and the lack of fine particulate matter (average aerodynamic diameter of less than 2.5 μm; PM_2.5_) and ozone (O_3_) data from most Chinese cities further hinders the value of such studies [2]. According to the World Health Organization’s (WHO’s) air quality standards, the acceptable daily average concentrations of PM_2.5_ and PM_10_ (PM with an average aerodynamic diameter of less than 10 μm) are 25 μg/m^3^ and 50 μg/m^3^, respectively, and the 8-h average concentration of O_3_ is 100 μg/m^3^ [4]. In China, even areas with relatively good air quality may not meet the WHO’s air quality standard.

Previous studies have shown that air pollution and outpatient visits are likely associated with respiratory and circulatory diseases [5, 6]. High pollutant concentrations can even increase the daily cardiovascular/respiratory death rates [7]. For example, NO_2_ may cause lung cancer [8], PM has been associated with increased blood pressure (BP), and a certain concentration of ozone has been associated with decreased BP [9]. There is an association between PM_2.5_ and inflammation [10]. PM_2.5_ can also increase the incidence of various respiratory and circulatory diseases [11, 12]. Even low air pollution concentrations can increase the risk of emergency department visits [13]. One study in an area with a low level of air pollution found that interquartile range (IQR) increases in PM_2.5_, PM_10_, NO_2_ and O_3_ were related to increases in outpatient visits for respiratory conditions [5]. A study that lasted approximately 7 years and considered over 4 million emergency department visits in 31 hospitals showed an association between cardiovascular disease and ambient pollutant levels [14]. Another study conducted over 17 years in Canada reported that ozone is highly associated with circulatory hospitalizations [15]. Some studies have suggested that low-level PM exposure could cause an increased excess risk (ER) of circulatory outpatient visits [11, 16]. Overall, even if the air quality is good in some regions, the effects of air pollution cannot be ignored.

Similar studies may obtain different outcomes because of differences in pollution concentrations and components and different population age structures and sensitivities among different regions [3]. In particular, regarding the concentration of air pollutants, the association between air pollution and health effects in areas with poor air quality was lower than that in areas with good air quality [2, 17]. Describing the effects of air pollutants via comparisons with analyses of data from other regions is inappropriate; thus, research on the association between air pollutants and outpatient visits is necessary to understand the effects of local air pollution.

Modeling is particularly important in such studies. A single air pollutant model is not sufficient, and comprehensive air pollutant models that consider synergistic effects are essential for studying the association between air pollutants and outpatient visits [18]. We conducted this study to analyze the associations between air pollutants and outpatient visits in Fuzhou using a comprehensive air pollutant model that considers the synergistic effects of different air pollutants, and a total single air pollutant model, a seasonal model (examining the cold season and warm season) and a double pollutant model were constructed.

## Methods

### Data collection

Our daily air pollution monitoring data were based on 3-year data collection from 1 January 2016 to 31 December 2018 from seven air pollution monitoring stations of the Fuzhou Environmental Monitoring Center Station, and daily meteorological monitoring data were collected through daily monitoring by the Fuzhou Meteorological Bureau, which is part of the nationwide network of monitoring stations and strictly implements relevant national technical requirements. The indicators of air pollution included nitrogen dioxide (NO_2_), daily maximum 8-h mean ozone (O_3_-8h), PM_10_ and PM_2.5_. The meteorological indicators included air pressure (AP), relative humidity (RH) and temperature (T). Values of O_3_-8h, PM_10_ and PM_2.5_ were evaluated based on WHO air quality standards (100, 50 and 25 μg/m^3^, respectively), and NO_2_ was evaluated based on the China class I air quality standard (80 μg/m^3^). The daily outpatient visits of Jianxin Hospital and Kongjun Hospital were collected by the Fuzhou Center for Disease Control and Prevention, which has been part of the Fuzhou health monitoring network for 3 consecutive years and was subject to strict quality control according to requirements. We identified diseases according to their International Classification of Diseases, 10th edition (ICD-10) codes (J00-J99 for respiratory diseases and I00-I99 for circulatory diseases). Among the J00-J99 codes, J00-J06 and J30-J39 represent upper respiratory diseases and J20-J22, J40-J47, and J85-J86 represent lower respiratory diseases.

In the analysis of the association between outpatient visits and air pollutants, there were meteorological factors and natural fluctuations in daily events over the course of a week that we needed to account for. We only collected the total number of outpatient visits that was not contain any individual or patient data. Ethics approval and consent to participate were unnecessary for the present study in accordance with the IRB of Fuzhou Center for Disease Control and Prevention.

### Statistical analysis

In this study, a generalized linear model (GLM) was used to analyze the association between outpatient visits and the studied air pollutants. The GLM with a time-series regression analysis was based on a Poisson distribution. We introduced meteorological parameters, including T (°C) and RH (%). Because the relationship between meteorology and health is generally nonlinear, we used a natural smoothing spline function to control for this nonlinear hybrid effect. We used 3 degrees of freedom for T and RH [7, 18, 19]. The natural spline (ns) function of date was also used in the GLM to address nonlinear trends, sequence correlations and the number of events per day on the time axis. The day of the week (DOW) was considered in this model to control for the natural fluctuation trends over a week. The degrees of freedom (df) for date were 7 df per year [20, 21]. The model is as follows [22, 23]:

$$ \log E(Yt)=\beta \mathrm{Zt}+\mathrm{ns}\left(\mathrm{time},7\ast 3\right)+ DOW+ ns\left( Xt,3\right)+\operatorname{int} ercept $$

where E (Yt) is the expected value of the number of outpatient visits on day t; Zt is the pollutant concentration on day t; β is the exposure-response coefficient; ns () is the natural smoothing spline function; df is the degrees of freedom; time is the calendar time variation; DOW is the weekly variation; and Xt is the meteorological factor.

The study analyzed the ER of outpatient visits associated with air pollutants and included a total single air pollutant model, seasonal model (cold period and warm season) ozone model (exceeding 100 μg/m^3^ of ozone), and double pollutant model. The double air pollutant model considered data from lag0. The seasonal model was divided into a cool period and warm period according to the monthly mean T. The months in which the monthly mean T exceeded 20 °C were considered the warm period (April–October). Otherwise, the months were considered the cool period (November–March of the following year). The ozone model exceeding 100 μg/m^3^ did not introduce the ns function of date and DOW because of discontinuity. The model is as follows:

$$ \log E(Yt)=\beta \mathrm{Zt}+ ns\left( Xt,3\right)+\operatorname{int} ercept $$

where E (Yt) is the expected value of the number of outpatient visits on day t; Zt is the pollutant concentration on day t; β is the exposure-response coefficient; ns () is the natural smoothing spline function; and df is the degrees of freedom. We also conducted a Wilcoxon paired test to identify the significance of the relationship in different seasons. The mgcv package of the R 3.5.1 statistical software platform was used for calculating and painting.

## Results

### Descriptive analyses

Table 1 shows that during the study, the mean pollutant concentrations were 27.38 μg/m^3^ for NO_2_, 89.60 μg/m^3^ for daily O_3_-8h, 26.07 μg/m^3^ for PM_2.5_, and 49.68 μg/m^3^ for PM_10_. During the study, the O_3_-8h concentration exceeded 100 μg/m^3^ for a total of 390 days, the NO_2_ concentration exceeded 80 μg/m^3^ for a total of 0 days, the PM_2.5_ concentration exceeded 25 μg/m^3^ for a total of 509 days, and the PM_10_ concentration exceeded 50 μg/m^3^ for a total of 478 days. The mean daily average T, RH and AP were 21.54 °C, 72% and 1010 hpa, respectively.

**Table 1:** Statistical summary of daily air pollutants, meteorological factors and outpatient visits in Fujian, China, 2016–2018

Variable	Mean ± SD	Minimum	Percentile			Maximum
			25th	50th	75th	
**Total**						
Respiratory disease	82 ± 32	1	60	83	104	180
Upper respiratory disease	49 ± 20	0	35	49	63	113
Lower respiratory disease	16 ± 8	0	10	16	22	47
Circulatory disease	174 ± 83	0	105	191	231	382
NO_2_ (μg/m^3^)	27.38 ± 11.18	3.83	19.55	25.14	33.14	79.57
O_3_-8h (μg/m^3^)	89.60 ± 33.94	16.71	64.02	87.36	112.00	208.43
PM_2.5_ (μg/m^3^)	26.07 ± 13.14	2.43	16.57	23.86	32.71	83.57
PM_10_ (μg/m^3^)	49.68 ± 22.62	7.43	33.33	46.49	63.89	167.57
Temperature (°C)	21.54 ± 7.08	2.60	15.60	22.00	28.00	32.80
Relative humidity (%)	72 ± 11	33	65	72	79	99
Air pressure (hpa)	1010 ± 8	983	1003	1009	1016	1034
**Cold season**						
Respiratory disease	91 ± 34	1	70	96	114	166
Upper respiratory disease	55 ± 22	0	43	56	70	106
Under respiratory disease	18 ± 9	0	12	19	25	46
Circulatory disease	175 ± 84	0	108	195	236	367
NO_2_ (μg/m^3^)	33.11 ± 11.55	10.43	24.57	31.72	41.34	79.57
O_3_-8h (μg/m^3^)	74.21 ± 26.59	16.71	53.66	73.72	92.97	168.14
PM_2.5_ (μg/m^3^)	30.42 ± 14.44	2.43	19.86	28.14	38.50	82.14
PM_10_ (μg/m^3^)	52.28 ± 23.48	7.43	33.72	50.79	68.54	134.14
Temperature (°C)	14.66 ± 4.11	2.60	11.60	14.30	14.30	24.70
Relative humidity (%)	72 ± 12	33	63	73	80	98
Air pressure (hpa)	1017 ± 6	1001	1013	1016	1021	1034
**Warm season**						
Respiratory disease	76 ± 29	11	56	77	95	180
Upper respiratory disease	45 ± 18	5	33	44	56	113
Under respiratory disease	15 ± 8	0	10	15	20	47
Circulatory disease	173 ± 81	13	103	189	229	382
NO_2_ (μg/m^3^)	23.32 ± 8.92	3.83	17.74	22.00	27.20	73.29
O_3_-8h (μg/m^3^)	100.48 ± 34.39	30.00	75.04	99.24	124.14	208.43
PM_2.5_ (μg/m^3^)	22.99 ± 11.17	4.86	15.14	21.00	28.86	83.57
PM_10_ (μg/m^3^)	47.84 ± 21.82	10.29	33.04	44.14	60.03	167.57
Temperature (°C)	26 ± 4	14.50	23.53	27.10	27.10	32.80
Relative humidity (%)	72 ± 11	41	65	72	79	99
Air pressure (hpa)	1005 ± 6	983	1001	1004	1008	1022
**O**_3_**concentrations exceeding 100 μg/m**^**3**^						
Respiratory disease	82 ± 30	12	61	84	102	163
Upper respiratory disease	49 ± 19	7	36	47	62	103
Lower respiratory disease	17 ± 8	0	11	17	23	47
Circulatory disease	181 ± 81	18	115.5	194.5	238.75	382
NO_2_ (μg/m^3^)	25.16 ± 7.56	10.14	20.14	23.745	28.2825	56.14
O_3_-8h (μg/m^3^)	126.36 ± 20.89	100.14	109.86	121.71	136.66	208.43
PM_2.5_ (μg/m^3^)	29.81 ± 10.97	6.43	21.74	27.86	35.57	70.43
PM_10_ (μg/m^3^)	59.81 ± 19.55	15.29	45.895	57.43	70.86	164.14
Temperature (°C)	24.36 ± 6.46	8.5	19.75	26	26	32.8
Relative humidity (%)	66 ± 9	41	60	66	72	96
Air pressure (hpa)	1007 ± 7	992	1002	1006	1013	1031

### Association between air pollution and meteorological factors

**Fig. 1:**
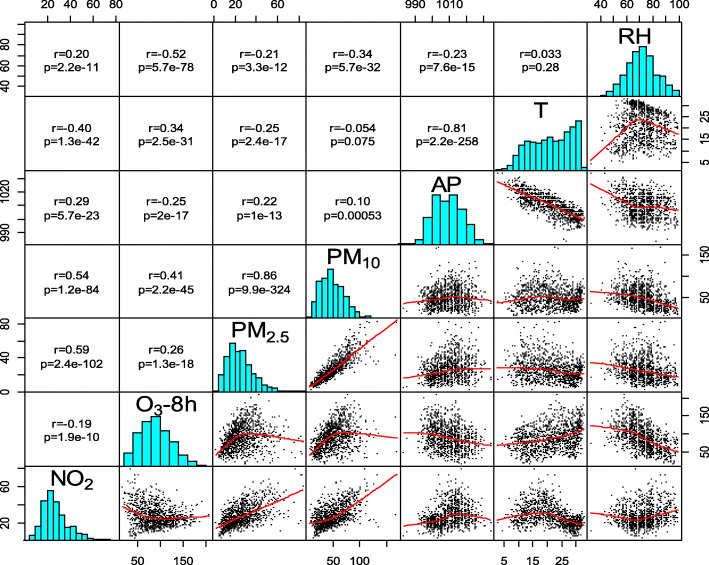
Spearman correlations between air pollutants and meteorological factors from 2016 to 2018. RH, relative humidity; T, temperature; AP, air pressure; PM_10_, particulate matter with an aerodynamic diameter less than 10 μm^− 3^; PM_2.5_, particulate matter with an aerodynamic diameter less than 2.5 μm^− 3^; O_3_-8h, daily maximum 8-h mean ozone; NO_2_, nitrogen dioxide

Figure 1 shows that except for T and PM_10_, meteorological factors were significantly correlated with air pollutants. RH was positively correlated with NO_2_ and negatively correlated with O_3_-8h, PM_2.5_ and PM_10_. T was positively correlated with O_3_-8h and negatively correlated with NO_2_, PM_2.5_ and PM_10_. AP was positively correlated with PM_10_, NO_2_ and PM_2.5_ and negatively correlated with O_3_-8h.

### Time series distribution of air pollutants and outpatient visits

**Fig. 2:**
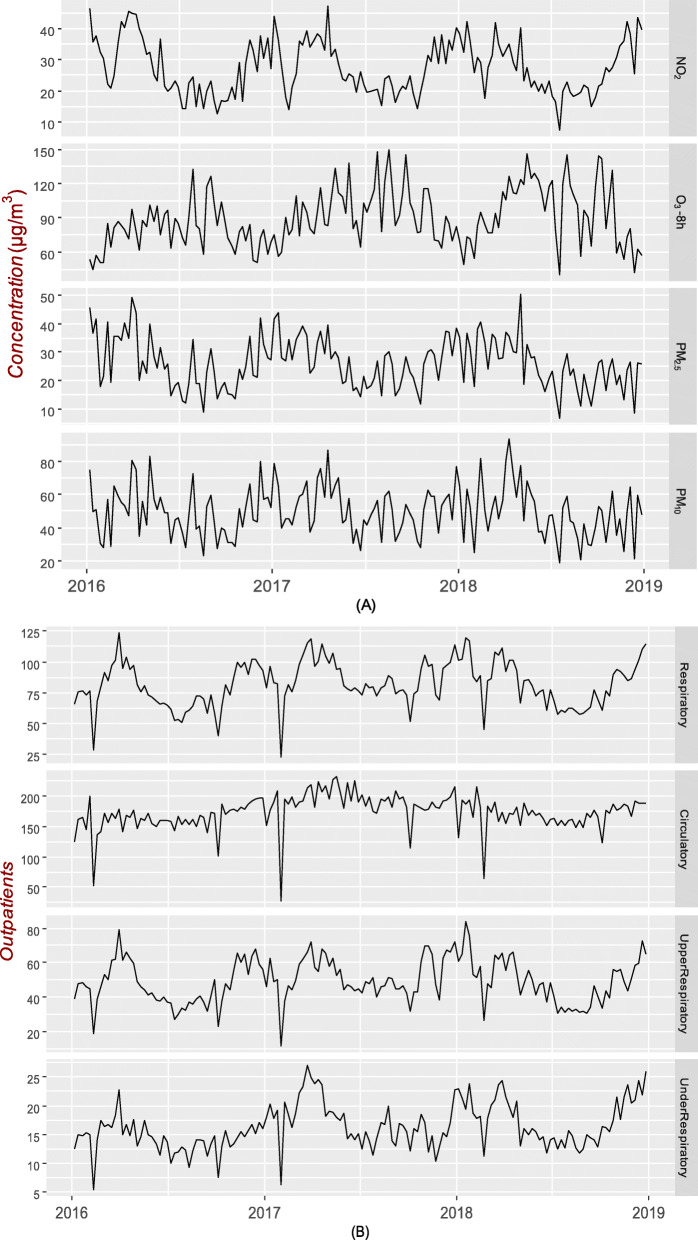
Time series distribution of air pollutants and outpatient visits. Time series graphs of weekly air pollutants (**a**) and outpatient visits (**b**) for total respiratory, lower respiratory, upper respiratory and circulatory diseases

Figure 2a shows that NO_2_, PM_10_ and PM_2.5_ had higher concentrations during the cold season than during the warm season while O_3_-8h had a higher concentration during the warm season than the cold season. Figure 2b shows that respiratory diseases, including upper and lower respiratory diseases, resulted in more outpatient visits during the cold season than during the warm season.

### Association between air pollutants and outpatient visits

**Fig. 3:**
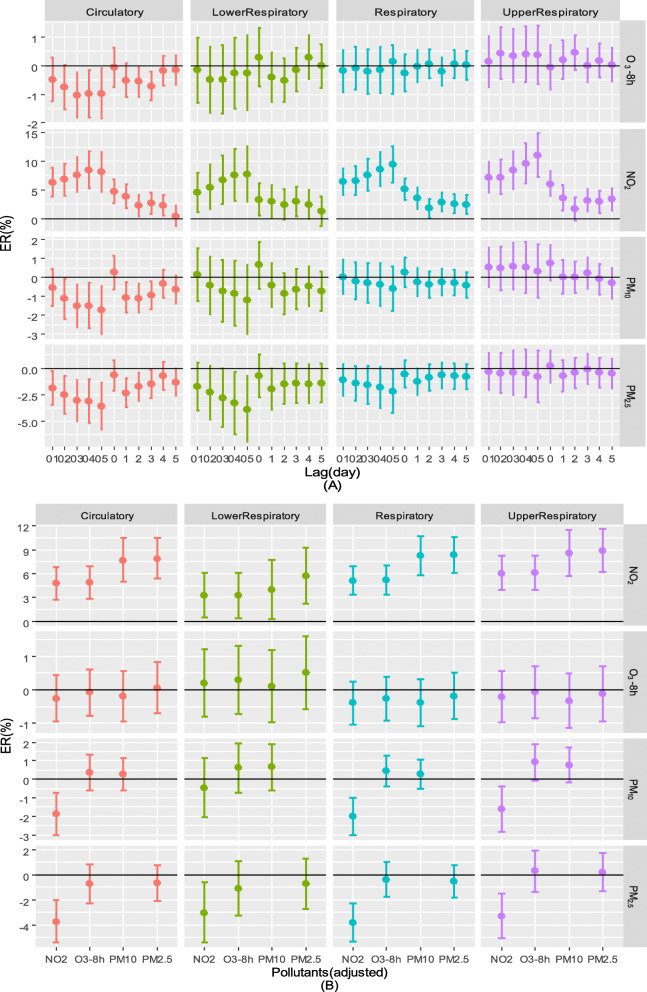
ERs of outpatient visits for total respiratory, lower respiratory, upper respiratory and circulatory diseases. Single air pollutant models (**a**) and double air pollutant models (**b**). The ER (%) of the y-axis indicates that a 10 μg/m^3^ increase in the concentration of air pollutants increases outpatient visits; “01, 02, 03, 04, 05” represent the cumulative lag effect

In Fig. 3a, the single air pollutant model shows that NO_2_ had a significant effect on the ER of total respiratory, lower respiratory, upper respiratory and circulatory diseases. Moreover, Table S1 also provides the single-day lag effect that was most obvious at lag0 and increased by 5.11% (95% CI: 3.31–6.95%) for total respiratory visits, 6.04% (95% CI: 3.91–8.21%) for upper respiratory visits, 3.23% (95% CI: 0.46–6.08%) for lower respiratory visits, and 4.75% (95% CI: 6.81–2.73%) for circulatory outpatient visits. The cumulative lag effect was the most obvious at lag0–5 and increased by 9.43% (95% CI: 6.31–12.65%) for total respiratory disease, 10.96% (95% CI: 7.22–14.84%) for upper respiratory disease, 7.69% (95% CI: 2.96–12.64%) for lower respiratory disease, and 8.14% (95% CI: 4.74–11.65%) for circulatory diseases.

As shown in Fig. 3b and Table S2, after adjusting for the three other air pollutants, the ER of O_3_-8h did not obviously change. After adjusting for PM_2.5_ and PM_10_, NO_2_ increased greatly. Because of the possible collinearity of PM_10_ and PM_2.5_, we did not introduce them into our model, although after adjusting for NO_2_, the ERs of PM_10_ and PM_2.5_ decreased.

**Fig. 4:**
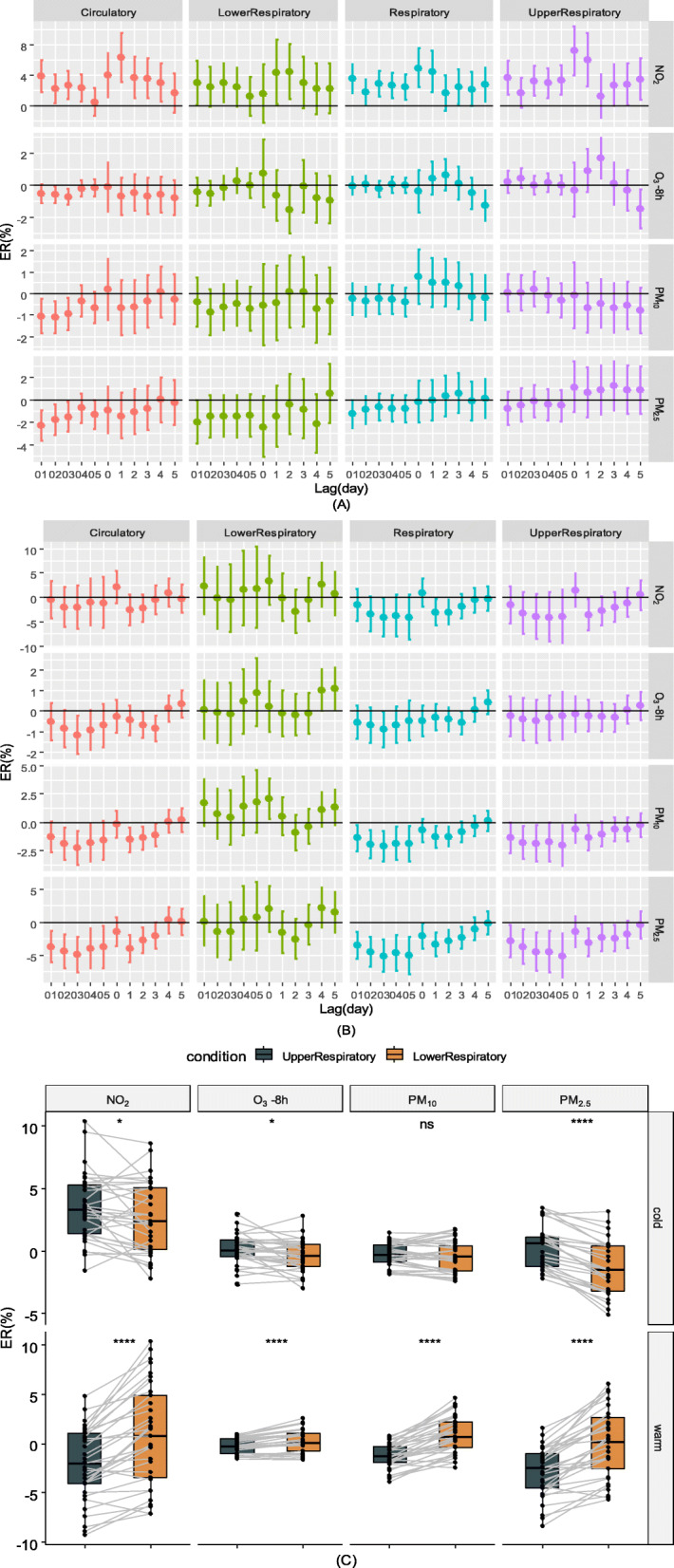
ERs of outpatient visits for total respiratory, lower respiratory, upper respiratory and circulatory diseases in the different periods. Cold period (**a**), warm period (**b**) and the Wilcoxon paired test of ERs between the upper respiratory and lower respiratory periods in different periods (**c**). (*p* > 0.05: ‘ns’; 0.01 < *p* < 0.05: ‘^*^’; 0.001 < *p* < 0.01: ‘^**^’; 0.0001 < *p* < 0.001:‘^***^’; *p* < 0.0001: ‘^****^’)

Figure 4a shows the association between the different air pollutants and outpatient visits during the cold seasons. During the cold season, NO_2_ appeared to have an obvious effect, although its effect was less than that during the warm season. Eight-hour O_3_ had a significant impact on outpatient visits for upper respiratory diseases at lag2 during the cold season, and the ER was 1.68% (2.94–0.44%), as also shown in Table S3.

Figure 4b shows the association between the different air pollutants and outpatient visits during the warm season. During this period, NO_2_ had no significant association with outpatient visits. Eight-hour O_3_ had a significant effect on outpatient visits for lower respiratory conditions at lag4 (1.02, 95% CI: 0.032–2.02%) and lag5 (1.10, 95% CI: 0.11–2.11%), and PM_10_ had a significant impact on outpatient visits for upper respiratory conditions at lag0 (2.05%, CI: 0.27–3.86%) (see Additional file 1: Table S4). Figure 4c shows the results of the Wilcoxon paired test for lower and upper respiratory diseases in different seasons that was used to test for significance. We calculated the circulatory effects of air pollutants when the daily T was higher and lower than 30 °C during the warm season, and we found that heat posed higher risks of circulatory disease, although the difference was not significant (see Additional file 1: Fig. S1).

**Fig. 5:**
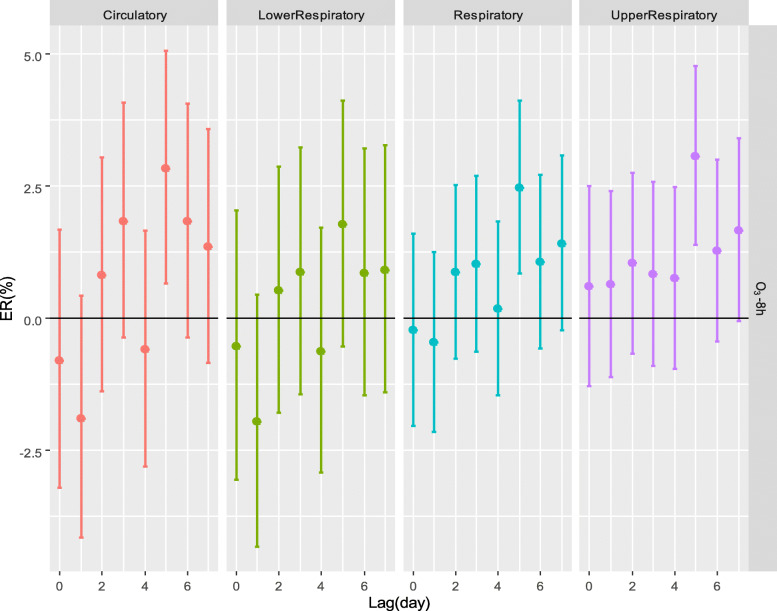
ERs of outpatient visits when the O_3_-8h concentration was over 100 μg/m^3^

Figure 5 and Table S5 show that when the concentration of O_3_-8h was higher than 100 μg/L, there was a positive effect on circulatory [lag5, ER (95% CI): 2.83% (0.65–5.06%)], respiratory [lag5, ER (95% CI): 2.47% (0.85–4.11%)] and upper respiratory [lag5, ER (95% CI): 3.06% (1.38–4.77%)] outpatient visits.

## Discussion

In this study, NO_2_ presented a more obvious effect than the other three air pollutants in Fuzhou. We explored the relationship between air pollutants and outpatient visits for different diseases and in different seasons. During the cold season, there were more outpatient visits for respiratory, upper respiratory and circulatory diseases in association with the effects of O_3_-8h, PM_2.5_ and PM_10_ than during the warm season; however, during the warm season, there were more outpatient visits for lower respiratory diseases in association with those three air pollutants than there were during the cold season. In the double pollutant model, after adjusting for NO_2_, the effects of the other three air pollutants decreased. After adjusting for PM_2.5_, PM_10_ showed a significant effect. After adjusting for the other three air pollutants, the ER of O_3_-8h changed only slightly. Different air pollutants presented different effects because of different conditions.

Our study showed the association between meteorological factors and the air pollutants NO_2_, O_3_-8h, PM_10_ and PM_2.5_. We found that the action of AP, RH and T caused high concentrations of air pollutants. Other studies have shown that meteorological factors have an effect on the concentrations of air pollutants, which is similar to the findings of our study [24, 25]. RH is known to increase haze, possibly because RH is positively correlated with NO_2_, which converts from the gas phase of NO_x_ to the particulate phase under relatively low-visibility conditions [26]. We did not find a positive association between PM and RH in the Spearman correlation model, although in the contour plot, the correlation between RH and PM_10_ and PM_2.5_ first increased and then decreased at a certain AP and T. The joint action of meteorological factors had seemingly obvious effects on PM. O_3_ had a positive association with T and a negative association with RH because sunshine might be the main promoter of O_3_ because O_3_ is enhanced by photochemical factors and RH can affect sunshine duration [27]. Meteorological factors can influence air pollution, thereby impacting health. Therefore, meteorological factors were introduced into the GLM. The time series diagram shows that during the cold season, all the air pollutants except ozone had higher concentrations than during the warm season. This discrepancy is due to the negative association between T and air pollutants and the positive association between AP and air pollutants other than ozone. In addition to meteorological factors, emissions also increase pollutant concentrations [28]. During the cold season in Fuzhou, heating is provided by lighting fires rather than using coal, which leads to increased PM emissions. The time series diagram also shows that there were more outpatient visits for respiratory diseases, including upper and lower respiratory diseases, during the cold season than the warm season, which may be different from the findings for other regions; for example, the spring dust storm season in Lanzhou may increase emergency room visits for respiratory diseases [29].

The GLM reflected different aspects, including the total situation, different seasons and double air pollutants. Because of the significant effects of the different air pollutants, we conducted a comprehensive study to evaluate the ERs of NO_2_, O_3_, PM_2.5_ and PM_10_ [30, 31]. In the overall model, we found that NO_2_ had a more obvious effect than the other three air pollutants on the ER of outpatient visits, especially considering the cumulative lag effect. Some studies also found that NO_2_ was strongly associated with hospital admissions for both respiratory and cardiovascular diseases [32, 33]. In China, the Sixth National Population Census showed that coastal areas had become old-age societies and a systematic review and meta-analysis reported that the effect of NO_2_ exhibited regional differences because of differences in the proportions of elderly people with increased susceptibility to NO_2_, which may be the cause of the high ER associated with NO_2_ [34]. Even when air quality is not poor, the elderly may still be susceptible to air pollutants.

However, when we examined the results according to season, the effect of NO_2_ was less significant than that of the total situation. Moreover, the ER of NO_2_ was lower during the warm season than the cold season and lost all significance during the warm season. In addition to T, the concentrations of air pollutants differed between the cold season and warm season. During the cold season, the concentration of NO_2_ was 33.11 μg/m^3^, while during the warm season, it was 23.32 μg/m^3^. There is a dose-dependent relationship between pulmonary injuries and ambient NO_2_ [35], although for circulatory injuries, such research is lacking. Interestingly, NO_2_, O_3_, PM_2.5_ and PM_10_ had similar results in terms of their influence on outpatient visits for upper and lower respiratory diseases. Generally, PM_10_ has a greater impact on the upper respiratory tract than the lower respiratory tract, while PM_2.5_ and O_3_ exhibit the opposite effect. During the cold season, the increase in outpatient visits for upper respiratory disease was greater than that during the warm season, while the opposite results were observed for lower respiratory disease-related visits except in the case of NO_2_. Nitrogen dioxide, ozone and PM_2.5_ caused more ERs for upper respiratory-related outpatient visits than for lower respiratory-related outpatient visits during the cold period, whereas nitrogen dioxide, ozone, PM_2.5_ and PM_10_ caused more ERs for lower respiratory-related outpatient visits during the warm season. T and AP were 14.66 °C and 1016.65 hPa during the cold season, respectively, and 26.40 °C and 1004.66 hPa during the warm season, respectively. Some studies reported that low AP and warm T increased susceptibility to respiratory-related diseases [36, 37]. A study pointed out that a greater diurnal T range caused more outpatient visits for the common cold [38]. Similarly, greater T change affects the number of hospital admissions for chronic obstructive pulmonary disease [39]. Fuzhou often experiences a high diurnal T range during cold periods. However, PM and O_3_ had greater effects on upper respiratory-related outpatient visits during the cold season and lower respiratory-related outpatient visits during the warm season, which was possibly because the depths of the respiratory tract that pollutants are able to reach are impacted by T and AP; however, this theory needs further study. Regarding circulatory diseases, in our study, we found that during the cold season, air pollutants increased the number of outpatient visits for circulatory diseases, and previous studies have presented similar outcomes [33, 40]. However, a study conducted over a 17-year period in Canada reported that 1-day lagged ozone had a greater association with the three examined circulatory hospitalization causes (ischemic heart disease, other heart disease and cerebrovascular disease) during the warm season than during the cold season [15]. A study in Hong Kong reported that PM and NO_2_ increased emergency hospital admissions during the warm season [41]. During our study, increased concentrations of PM and NO_2_ were observed during the cold season while an increased concentration of O_3_ was not. In addition to the increased concentrations of air pollutants, heat waves and other extreme high-T events were more likely to occur on low-T days, which may cause more outpatient visits for circulatory diseases [42]. We found that during the warm season of high Ts (> 30 °C), pollutants cause greater damage to the cardiovascular system than when T is less than 30 °C. Studies have reported that under high-T conditions, the risk of ozone-related cardiovascular death increases and PM has a greater impact on the cardiovascular system; thus, T and pollutants may have a synergistic effect on cardiovascular disease [18, 43]. However, a study in low-pollution areas found that the effects of PM_2.5_ were more obvious during the cool season than the warm season [5].

We also conducted analyses of ozone concentrations exceeding 100 μg/m^3^ because ozone pollution is serious. The model with ozone exceeding 100 μg/m^3^ did not introduce the ns function of date and DOW because of discontinuities. Ozone exceeded 100 μg/m^3^ for a total of 390 days during the study period (total study period, 1096 days) and a total of 315 days during the warm season. The warm season model showed that high ozone levels had a significant effect on respiratory outpatient visits at lag4 and lag5. The time at which the significant effect appeared was the same in the warm period model and the ozone exceeding 100 μg/m^3^ model, although the predominant diseases were different, which may be related to the increased concentration of O_3_ in the 100 μg/m^3^ ozone model [O_3_-8h average (standard O_3_-8h concentration model): 126.36 μg/m^3^ vs O_3_-8h average (warm season average): 100.48 μg/m^3^].

In the double model, after adjusting for PM_2.5_, NO_2_ and O_3_-8h presented increased ERs at lag0. In contrast, after adjusting for NO_2,_ the other three pollutants, especially PM (PM_2.5_ and PM_10_), presented decreased ERs. There was a strong correlation between PM and NO_2_. The ER of ozone did not fluctuate considerably after adjusting for the three other pollutants. The interaction between PM and NO_2_ was strong, and the effect of O_3_-8h was independent. Previous studies also found a strong correlation between PM and gaseous air pollution except for O_3_, which did not change much after the other air pollutants were added to the model [6, 18]. Some studies on mechanics noted that inflammation, oxidative stress, changes in systemic coagulation functioning and reduced cardiac autonomic control occurred after exposure to gaseous air pollutants and PM [44, 45], which may trigger respiratory and cardiovascular events as well as high concentrations of air pollutants (except O_3_-8h) during the same period (the cold season). These factors may cause high correlations among air pollutants. Therefore, it is difficult to evaluate the independent effects of PM or NO_2_ because of their high correlations [17].

Several limitations affected this study. In coastal areas, ozone pollution is more serious than PM and NO_2_ pollution, although in this study, NO_2_ increased the number of outpatient visits. Eight-hour O_3_ and NO_2_ are related to photochemical smog, and they promote one another; thus, they may exhibit joint action. However, we could not find obvious interactions in the double model; therefore, further research is required. PM_2.5_ increased the outpatient visit risk rate in many studies, even in areas with better air quality than Fuzhou, which may indicate that there are regional differences in the effect of PM_2.5_ exposure in China [46]. In our study, we did not observe a significant effect of PM_2.5_. If we stratify the results by different ages and diseases, we may obtain significant outcomes. Overall, our study provided a comprehensive analysis of the association between air pollutants and outpatient visits. In some comprehensive studies of large cohorts in other regions, even low exposure to air pollutants can have health effects [47, 48]. However, there is a lack of studies on the association between specific respiratory and circulatory diseases and different air pollutants. The effects observed in this study were short-term effects, and studies of long-term effects still need to be conducted in coastal areas of China.

## Conclusions

An association was observed between air pollutants and respiratory and circulatory outpatient visits. During the cold season, the ER of NO_2_ was higher than that during the warm season for both respiratory- (both upper and lower) and circulatory-related outpatient visits. However, during the cold season, O_3_-8h, NO_2_ and PM_2.5_ had greater ERs for upper respiratory-related outpatient visits than for lower respiratory-related outpatient visits, and during the warm season, O_3_-8h, NO_2_, PM_10_ and PM_2.5_ had greater ERs for lower respiratory-related outpatient visits. In the double air pollutant model, PM and NO_2_ showed a high correlation.

## Supplementary information

**Additional file 1 Table S1-S4.** Percentage changes with 95% CIs for outpatient visits for respiratory and circulatory diseases according to air pollutants in different models.**Table S5.** Percentage changes with 95% CIs for outpatient visits for respiratory and circulatory diseases according to air pollutants in the model with ozone exceeding 100 μg/m^3^. **Fig. S1.** Percentage changes with 95% CIs for outpatient visits for circulatory diseases under conditions over 30 °C and under 30 °C (A). The Wilcoxon paired test was used to compared the ERs for circulatory outpatient visits between conditions over and under 30 °C (B).

## Data Availability

The data belongs to the Fuzhou Center for Disease Control and Prevention, and has obtained permission from the center. Moreover, data are available from the corresponding author (lby@mail.fjmu.edu.cn and sanny77@sina.com) on reasonable request.

## Abbreviations

AP: Air pressure
DOW: Day of the week
O_3_-8h: Daily maximum 8-h mean ozone
ERs: Excess risks
df: Degrees of freedom
GLM: Generalized linear model
ICD-10: 10th edition of the International Classification of Diseases
NO_2_: Nitrogen dioxide
ns: Natural spline
PM_10_: Particulate matter with an aerodynamic diameter less than 10 μm^− 3^
PM_2.5_: Particulate matter with an aerodynamic diameter less than 2.5 μm^− 3^
RH: Relative humidity; and T: temperature
