# Supplementary material for: Temporal cross-correlations between air pollutants and outpatient visits for respiratory and circulatory system diseases in Fuzhou, China
Source: BMC Public Health. 2020 Jul 20;20:1131. doi: 10.1186/s12889-020-08915-y (PMC7370472; doi:10.1186/s12889-020-08915-y)
Supplement: Supplementary file 1 — Additional file 1 Table S1-S4. Percentage changes with 95% CIs for outpatient visits for respiratory and circulatory diseases according to air pollutants in different models.Table S5. Percentage changes with 95% CIs for outpatient visits for respiratory and circulatory diseases according to air pollutants in the model with ozone exceeding 100 μg/m3. Fig. S1. Percentage changes with 95% CIs for outpatient visits for circulatory diseases under conditions over 30 °C and under 30 °C (A). The Wilcoxon paired test was used to compared the ERs for circulatory outpatient visits between conditions over and under 30 °C (B). [file 12889_2020_8915_MOESM1_ESM.docx]

**Temporal cross-correlations between air pollutants and outpatient visits for respiratory and circulatory system diseases in Fuzhou, China**

| **Table S1** ERs of the single air pollutant models (total) | | | | | | | | | | | | | |
| --- | --- | --- | --- | --- | --- | --- | --- | --- | --- | --- | --- | --- | --- |
|  | lag (days) | ERs (%) | | | | | | | | | | | |
|  |  | NO_2_ | | | O_3_ | | | PM_2.5_ | | | PM_10_ | | |
|  |  | fit | high | low | fit | High | low | fit | high | low | fit | high | low |
| respiratory | 0 | 5.11 | 6.95 | 3.31 | -0.26 | 0.39 | -0.91 | -0.54 | 0.76 | -1.82 | 0.27 | 1.07 | -0.52 |
|  | 1 | 3.51 | 5.41 | 1.64 | -0.04 | 0.52 | -0.59 | -1.26 | 0.00 | -2.50 | -0.25 | 0.48 | -0.99 |
|  | 2 | 1.75 | 3.44 | 0.09 | 0.06 | 0.56 | -0.44 | -0.90 | 0.33 | -2.10 | -0.37 | 0.33 | -1.06 |
|  | 3 | 2.84 | 4.51 | 1.21 | -0.22 | 0.27 | -0.71 | -0.66 | 0.55 | -1.86 | -0.25 | 0.44 | -0.93 |
|  | 4 | 2.58 | 4.24 | 0.96 | 0.05 | 0.54 | -0.44 | -0.75 | 0.45 | -1.94 | -0.28 | 0.40 | -0.95 |
|  | 5 | 2.42 | 4.09 | 0.77 | 0.01 | 0.50 | -0.48 | -0.81 | 0.38 | -2.00 | -0.41 | 0.26 | -1.08 |
|  | 0~1 | 6.36 | 8.64 | 4.12 | -0.20 | 0.54 | -0.93 | -1.11 | 0.35 | -2.56 | 0.02 | 0.91 | -0.86 |
|  | 0~2 | 6.56 | 9.12 | 4.05 | -0.09 | 0.66 | -0.84 | -1.42 | 0.21 | -3.03 | -0.20 | 0.77 | -1.17 |
|  | 0~3 | 7.61 | 10.39 | 4.89 | -0.21 | 0.56 | -0.98 | -1.61 | 0.18 | -3.37 | -0.30 | 0.75 | -1.34 |
|  | 0~4 | 8.57 | 11.56 | 5.65 | -0.16 | 0.65 | -0.96 | -1.82 | 0.11 | -3.72 | -0.39 | 0.74 | -1.50 |
|  | 0~5 | 9.43 | 12.65 | 6.31 | 0.14 | 0.71 | -0.98 | -2.19 | -0.14 | -4.21 | -0.61 | 0.58 | -1.78 |
| upper respiratory | 0 | 6.04 | 8.21 | 3.91 | -0.08 | 0.70 | -0.85 | 0.18 | 1.71 | -1.33 | 0.77 | 1.71 | -0.17 |
|  | 1 | 3.60 | 5.86 | 1.39 | 0.20 | 0.87 | -0.46 | -0.76 | 0.73 | -2.22 | 0.03 | 0.90 | -0.84 |
|  | 2 | 1.65 | 3.65 | -0.32 | 0.44 | 1.04 | -0.16 | -0.45 | 1.00 | -1.88 | 0.03 | 0.85 | -0.80 |
|  | 3 | 3.16 | 5.12 | 1.23 | 0.00 | 0.59 | -0.59 | -0.09 | 1.35 | -1.50 | 0.21 | 1.03 | -0.59 |
|  | 4 | 2.92 | 4.88 | 1.00 | 0.17 | 0.76 | -0.41 | -0.44 | 0.98 | -1.84 | -0.09 | 0.71 | -0.88 |
|  | 5 | 3.32 | 5.31 | 1.38 | 0.03 | 0.62 | -0.56 | -0.51 | 0.91 | -1.90 | -0.31 | 0.49 | -1.10 |
|  | 0~1 | 7.15 | 9.87 | 4.49 | 0.13 | 1.01 | -0.74 | -0.32 | 1.42 | -2.02 | 0.53 | 1.59 | -0.51 |
|  | 0~2 | 7.17 | 10.23 | 4.20 | 0.42 | 1.33 | -0.47 | -0.48 | 1.46 | -2.38 | 0.47 | 1.63 | -0.68 |
|  | 0~3 | 8.40 | 11.73 | 5.17 | 0.34 | 1.27 | -0.59 | -0.42 | 1.71 | -2.50 | 0.58 | 1.83 | -0.66 |
|  | 0~4 | 9.54 | 13.12 | 6.07 | 0.39 | 1.36 | -0.58 | -0.53 | 1.77 | -2.78 | 0.53 | 1.87 | -0.79 |
|  | 0~5 | 10.96 | 14.84 | 7.22 | 0.37 | 1.39 | -0.64 | -0.78 | 1.68 | -3.18 | 0.32 | 1.74 | -1.08 |
| lower respiratory | 0 | 3.23 | 0.46 | 6.08 | 0.29 | 1.31 | -0.72 | -0.76 | 1.27 | -2.75 | 0.65 | 1.89 | -0.59 |
|  | 1 | 2.90 | 5.86 | 0.02 | -0.40 | 0.46 | -1.26 | -1.96 | 0.00 | -3.88 | -0.41 | 0.73 | -1.55 |
|  | 2 | 2.41 | 5.05 | -0.17 | -0.52 | 0.26 | -1.28 | -1.48 | 0.42 | -3.35 | -0.87 | 0.22 | -1.94 |
|  | 3 | 2.91 | 5.52 | 0.36 | -0.15 | 0.61 | -0.91 | -1.46 | 0.42 | -3.30 | -0.64 | 0.43 | -1.69 |
|  | 4 | 2.39 | 4.97 | -0.13 | 0.29 | 1.05 | -0.48 | -1.47 | 0.39 | -3.30 | -0.46 | 0.59 | -1.49 |
|  | 5 | 1.17 | 3.76 | -1.35 | -0.02 | 0.75 | -0.78 | -1.41 | 0.46 | -3.24 | -0.71 | 0.33 | -1.75 |
|  | 0~1 | 4.48 | 8.02 | 1.06 | -0.17 | 0.97 | -1.30 | -1.75 | 0.55 | -4.00 | 0.13 | 1.52 | -1.24 |
|  | 0~2 | 5.43 | 9.40 | 1.60 | -0.50 | 0.66 | -1.65 | -2.31 | 0.24 | -4.80 | -0.44 | 1.07 | -1.94 |
|  | 0~3 | 6.63 | 10.94 | 2.47 | -0.49 | 0.70 | -1.67 | -2.82 | -0.04 | -5.52 | -0.72 | 0.91 | -2.32 |
|  | 0~4 | 7.54 | 12.18 | 3.09 | -0.27 | 0.97 | -1.50 | -3.30 | -0.31 | -6.20 | -0.85 | 0.89 | -2.56 |
|  | 0~5 | 7.69 | 12.64 | 2.96 | -0.26 | 1.04 | -1.55 | -3.88 | -0.70 | -6.96 | -1.19 | 0.65 | -2.99 |
| circulatory | 0 | 4.75 | 6.81 | 2.73 | -0.08 | 0.61 | -0.77 | -0.69 | 0.75 | -2.10 | 0.26 | 1.14 | -0.62 |
|  | 1 | 3.82 | 5.93 | 1.75 | -0.52 | 0.07 | -1.11 | -2.32 | -0.94 | -3.68 | -1.06 | -0.25 | -1.86 |
|  | 2 | 2.17 | 4.04 | 0.33 | -0.56 | -0.04 | -1.09 | -1.77 | -0.43 | -3.08 | -1.10 | -0.34 | -1.85 |
|  | 3 | 2.63 | 4.48 | 0.82 | -0.72 | -0.21 | -1.23 | -1.50 | -0.18 | -2.80 | -0.94 | -0.20 | -1.68 |
|  | 4 | 2.28 | 4.10 | 0.49 | -0.18 | 0.34 | -0.70 | -0.74 | 0.57 | -2.04 | -0.34 | 0.39 | -1.07 |
|  | 5 | 0.42 | 2.23 | -1.36 | -0.16 | 0.36 | -0.68 | -1.32 | -2.61 | -0.01 | -0.66 | 0.08 | -1.39 |
|  | 0~1 | 6.31 | 8.86 | 3.83 | -0.49 | 0.28 | -1.25 | -1.86 | -0.25 | -3.45 | -0.56 | 0.42 | -1.53 |
|  | 0~2 | 6.77 | 9.62 | 4.00 | -0.76 | 0.03 | -1.53 | -2.54 | -0.76 | -4.29 | -1.13 | -0.07 | -2.19 |
|  | 0~3 | 7.60 | 10.69 | 4.61 | -1.03 | -0.23 | -1.83 | -3.07 | -1.13 | -4.97 | -1.52 | -0.38 | -2.64 |
|  | 0~4 | 8.43 | 11.73 | 5.22 | -0.99 | -0.16 | -1.81 | -3.10 | -1.02 | -5.15 | -1.49 | -0.28 | -2.69 |
|  | 0~5 | 8.14 | 11.65 | 4.74 | -0.98 | -0.11 | -1.84 | -3.60 | -1.38 | -5.77 | -1.73 | -0.45 | -2.99 |

| **Table S2** ERs of the double air pollutant models (lag0) | | | | | | | | | | | | | |
| --- | --- | --- | --- | --- | --- | --- | --- | --- | --- | --- | --- | --- | --- |
|  | Pollutants (adjusted) | ERs(%) | | | | | | | | | | | |
|  |  | NO_2_ | | | O_3_ | | | PM_2.5_ | | | PM_10_ | | |
|  |  | fit | high | low | fit | high | low | fit | high | low | fit | high | low |
| respiratory | PM_10_ | 8.23 | 10.73 | 5.78 | -0.39 | 0.31 | -1.08 | -3.4 | -0.95 | -5.78 |  |  |  |
|  | PM_2.5_ | 8.33 | 10.64 | 6.06 | -0.19 | 0.51 | -0.88 |  |  |  | 2.1 | 3.66 | 0.56 |
|  | NO_2_ |  |  |  | -0.39 | 0.25 | -1.03 | -3.82 | -2.3 | -5.33 | -2.02 | -0.99 | -3.04 |
|  | O_3_ | 5.18 | 7.01 | 3.37 |  |  |  | -0.4 | 0.99 | -1.78 | 0.44 | 1.29 | -0.4 |
| upper respiratory | PM_10_ | 8.55 | 11.5 | 5.67 | -0.34 | 0.49 | -1.15 | -3.23 | -0.37 | -6 |  |  |  |
|  | PM_2.5_ | 8.86 | 11.61 | 6.18 | -0.13 | 0.71 | -0.95 |  |  |  | 2.51 | 4.35 | 0.71 |
|  | NO_2_ |  |  |  | -0.22 | 0.55 | -0.98 | -3.32 | -5.09 | -1.52 | -1.62 | -2.83 | -0.41 |
|  | O_3_ | 6.07 | 8.25 | 3.94 |  |  |  | 0.27 | 1.91 | -1.35 | 0.91 | 1.91 | -0.09 |
| lower respiratory | PM_10_ | 3.94 | 7.71 | 0.29 | 0.11 | 1.2 | -0.96 | -6 | -2.29 | -9.57 |  |  |  |
|  | PM_2.5_ | 5.7 | 9.27 | 2.25 | 0.5 | 1.6 | -0.59 |  |  |  | 3.93 | 6.37 | 1.54 |
|  | NO_2_ |  |  |  | 0.2 | 1.22 | -0.81 | -3.04 | -0.61 | -5.41 | -0.47 | 1.15 | -2.07 |
|  | O_3_ | 3.19 | 6.05 | 0.41 |  |  |  | -1.13 | 1.06 | -3.27 | 0.6 | 1.93 | -0.72 |
| circulatory | PM_10_ | 7.66 | 10.47 | 4.92 | -0.19 | 0.56 | -0.94 | -4 | -1.25 | -6.67 |  |  |  |
|  | PM_2.5_ | 7.88 | 10.47 | 5.36 | 0.06 | 0.82 | -0.69 |  |  |  | 2.43 | 4.2 | 0.69 |
|  | NO_2_ |  |  |  | -0.26 | 0.43 | -0.95 | -3.75 | -2.05 | -5.42 | -1.88 | -0.73 | -3.02 |
|  | O_3_ | 4.83 | 6.9 | 2.8 |  |  |  | -0.74 | 0.83 | -2.28 | 0.35 | 1.32 | -0.6 |

| **Table S3** ERs of the single air pollutant models (cold period) | | | | | | | | | | | | | |
| --- | --- | --- | --- | --- | --- | --- | --- | --- | --- | --- | --- | --- | --- |
|  | lag(day) | ERs (%) | | | | | | | | | | | |
|  |  | NO_2_ | | | O_3_ | | | PM_2.5_ | | | PM_10_ | | |
|  |  | fit | high | low | Fit | high | Low | fit | high | low | fit | high | low |
| respiratory | 0 | 4.88 | 7.46 | 2.35 | -0.38 | 0.96 | -1.7 | -0.15 | 1.7 | -1.97 | 0.78 | 2.05 | -0.46 |
|  | 1 | 4.41 | 7.22 | 1.67 | 0.44 | 1.49 | -0.59 | -0.04 | 1.8 | -1.84 | 0.51 | 1.67 | -0.64 |
|  | 2 | 1.56 | 3.9 | -0.72 | 0.66 | 1.65 | -0.33 | 0.33 | 2.12 | -1.44 | 0.5 | 1.62 | -0.6 |
|  | 3 | 2.43 | 4.7 | 0.21 | 0.11 | 1.14 | -0.9 | 0.56 | 2.34 | -1.19 | 0.37 | 1.46 | -0.71 |
|  | 4 | 2.1 | 4.34 | -0.1 | -0.48 | 0.5 | -1.46 | -0.13 | 1.63 | -1.86 | -0.17 | 0.9 | -1.22 |
|  | 5 | 2.73 | 4.98 | 0.52 | -1.26 | -0.31 | -2.21 | 0.11 | 1.85 | -1.61 | -0.18 | 0.86 | -1.21 |
|  | 0~1 | 3.51 | 5.41 | 1.64 | -0.04 | 0.52 | -0.59 | -1.26 | 0 | -2.5 | -0.25 | 0.48 | -0.99 |
|  | 0~2 | 1.75 | 3.44 | 0.09 | 0.06 | 0.56 | -0.44 | -0.9 | 0.33 | -2.1 | -0.37 | 0.33 | -1.06 |
|  | 0~3 | 2.84 | 4.51 | 1.21 | -0.22 | 0.27 | -0.71 | -0.66 | 0.55 | -1.86 | -0.25 | 0.44 | -0.93 |
|  | 0~4 | 2.58 | 4.24 | 0.96 | 0.05 | 0.54 | -0.44 | -0.75 | 0.45 | -1.94 | -0.28 | 0.4 | -0.95 |
|  | 0~5 | 2.42 | 4.09 | 0.77 | 0.01 | 0.5 | -0.48 | -0.81 | 0.38 | -2 | -0.41 | 0.26 | -1.08 |
| upper respiratory | 0 | 7.12 | 10.38 | 3.96 | -0.3 | 1.4 | -1.97 | 1.12 | 3.41 | -1.12 | -0.1 | 1.44 | -1.61 |
|  | 1 | 5.95 | 9.5 | 2.51 | 0.91 | 2.25 | -0.4 | 0.65 | 2.91 | -1.56 | -0.67 | 0.5 | -1.84 |
|  | 2 | 1.2 | 4.07 | -1.6 | 1.68 | 2.94 | 0.44 | 0.87 | 3.07 | -1.29 | -0.45 | 0.68 | -1.57 |
|  | 3 | 2.62 | 5.4 | -0.09 | 0.08 | 1.41 | -1.23 | 1.23 | 3.41 | -0.91 | -0.66 | 0.49 | -1.8 |
|  | 4 | 2.7 | 5.47 | 0.01 | -0.31 | 0.94 | -1.55 | 0.89 | 3.05 | -1.22 | -0.56 | 0.55 | -1.66 |
|  | 5 | 3.44 | 6.19 | 0.76 | -1.48 | -0.27 | -2.67 | 0.83 | 2.96 | -1.25 | -0.79 | 0.29 | -1.86 |
|  | 0~1 | 3.6 | 5.86 | 1.39 | 0.2 | 0.87 | -0.46 | -0.76 | 0.73 | -2.22 | 0.03 | 0.9 | -0.84 |
|  | 0~2 | 1.65 | 3.65 | -0.32 | 0.44 | 1.04 | -0.16 | -0.45 | 1 | -1.88 | 0.03 | 0.85 | -0.8 |
|  | 0~3 | 3.16 | 5.12 | 1.23 | 0 | 0.59 | -0.59 | -0.09 | 1.35 | -1.5 | 0.21 | 1.03 | -0.59 |
|  | 0~4 | 2.92 | 4.88 | 1 | 0.17 | 0.76 | -0.41 | -0.44 | 0.98 | -1.84 | -0.09 | 0.71 | -0.88 |
|  | 0~5 | 3.32 | 5.31 | 1.38 | 0.03 | 0.62 | -0.56 | -0.51 | 0.91 | -1.9 | -0.31 | 0.49 | -1.1 |
| lower respiratory | 0 | 1.53 | 5.42 | -2.22 | 0.71 | 2.83 | -1.36 | -2.41 | 0.35 | -5.1 | -0.55 | 1.37 | -2.43 |
|  | 1 | 4.3 | 8.64 | 0.13 | -0.63 | 0.97 | -2.2 | -1.49 | 1.26 | -4.17 | -0.45 | 1.3 | -2.16 |
|  | 2 | 4.38 | 8.03 | 0.85 | -1.53 | -0.01 | -3.02 | -0.43 | 2.28 | -3.06 | 0.07 | 1.76 | -1.59 |
|  | 3 | 2.97 | 6.4 | -0.35 | -0.05 | 1.59 | -1.66 | -0.83 | 1.82 | -3.41 | 0.07 | 1.71 | -1.54 |
|  | 4 | 2.12 | 5.48 | -1.13 | -0.78 | 0.76 | -2.29 | -2.11 | 0.5 | -4.66 | -0.72 | 0.88 | -2.29 |
|  | 5 | 2.2 | 5.55 | -1.04 | -0.92 | 0.56 | -2.38 | 0.54 | 3.18 | -2.02 | -0.35 | 1.22 | -1.89 |
|  | 0~1 | 2.9 | 5.86 | 0.02 | -0.4 | 0.46 | -1.26 | -1.96 | 0 | -3.88 | -0.41 | 0.73 | -1.55 |
|  | 0~2 | 2.41 | 5.05 | -0.17 | -0.52 | 0.26 | -1.28 | -1.48 | 0.42 | -3.35 | -0.87 | 0.22 | -1.94 |
|  | 0~3 | 2.91 | 5.52 | 0.36 | -0.15 | 0.61 | -0.91 | -1.46 | 0.42 | -3.3 | -0.64 | 0.43 | -1.69 |
|  | 0~4 | 2.39 | 4.97 | -0.13 | 0.29 | 1.05 | -0.48 | -1.47 | 0.39 | -3.3 | -0.46 | 0.59 | -1.49 |
|  | 0~5 | 1.17 | 3.76 | -1.35 | -0.02 | 0.75 | -0.78 | -1.41 | 0.46 | -3.24 | -0.71 | 0.33 | -1.75 |
| circulatory | 0 | 3.9 | 6.84 | 1.04 | -0.1 | 1.44 | -1.61 | -0.9 | 1.18 | -2.94 | 0.19 | 1.62 | -1.22 |
|  | 1 | 6.23 | 9.46 | 3.1 | -0.67 | 0.5 | -1.84 | -1.42 | 0.65 | -3.45 | -0.66 | 0.64 | -1.94 |
|  | 2 | 3.63 | 6.36 | 0.97 | -0.45 | 0.68 | -1.57 | -1.06 | 0.97 | -3.04 | -0.61 | 0.65 | -1.86 |
|  | 3 | 3.55 | 6.17 | 1 | -0.66 | 0.49 | -1.8 | -0.75 | 1.23 | -2.7 | -0.35 | 0.88 | -1.56 |
|  | 4 | 2.97 | 5.53 | 0.47 | -0.56 | 0.55 | -1.66 | 0.01 | 2.01 | -1.96 | 0.07 | 1.28 | -1.12 |
|  | 5 | 1.59 | 4.14 | -0.9 | -0.79 | 0.29 | -1.86 | -0.24 | 1.74 | -2.18 | -0.27 | 0.91 | -1.43 |
|  | 0~1 | 3.82 | 5.93 | 1.75 | -0.52 | 0.07 | -1.11 | -2.32 | -0.94 | -3.68 | -1.06 | -0.25 | -1.86 |
|  | 0~2 | 2.17 | 4.04 | 0.33 | -0.56 | -0.04 | -1.09 | -1.77 | -0.43 | -3.08 | -1.1 | -0.34 | -1.85 |
|  | 0~3 | 2.63 | 4.48 | 0.82 | -0.72 | -0.21 | -1.23 | -1.5 | -0.18 | -2.8 | -0.94 | -0.2 | -1.68 |
|  | 0~4 | 2.28 | 4.1 | 0.49 | -0.18 | 0.34 | -0.7 | -0.74 | 0.57 | -2.04 | -0.34 | 0.39 | -1.07 |
|  | 0~5 | 0.42 | 2.23 | -1.36 | -0.16 | 0.36 | -0.68 | -1.32 | -0.01 | -2.61 | -0.66 | 0.08 | -1.39 |

| **Table S4** ERs of the single air pollutant models (warm period) | | | | | | | | | | | | | |
| --- | --- | --- | --- | --- | --- | --- | --- | --- | --- | --- | --- | --- | --- |
|  | lag(day) | ERs(%) | | | | | | | | | | | |
|  |  | NO_2_ | | | O_3_ | | | PM_2.5_ | | | PM_10_ | | |
|  |  | fit | high | low | fit | High | low | fit | high | low | fit | high | low |
| respiratory | 0 | 0.89 | 3.81 | -1.95 | -0.48 | 0.24 | -1.2 | -2.08 | -0.14 | -3.99 | -0.73 | 0.31 | -1.76 |
|  | 1 | -3.07 | -0.32 | -5.74 | -0.31 | 0.34 | -0.95 | -3.36 | -1.57 | -5.12 | -1.28 | -0.31 | -2.24 |
|  | 2 | -3.06 | -0.52 | -5.54 | -0.41 | 0.17 | -1 | -2.88 | -1.15 | -4.57 | -1.26 | -0.36 | -2.15 |
|  | 3 | -1.88 | 0.67 | -4.37 | -0.58 | -0.02 | -1.14 | -2.34 | -0.62 | -4.04 | -0.85 | 0.04 | -1.74 |
|  | 4 | -0.56 | 1.98 | -3.04 | 0.07 | 0.64 | -0.51 | -1.02 | 0.69 | -2.71 | -0.34 | 0.55 | -1.23 |
|  | 5 | -0.3 | 2.27 | -2.79 | 0.41 | 0.99 | -0.16 | -0.12 | 1.62 | -1.83 | 0.1 | 1 | -0.78 |
|  | 0~1 | -1.59 | 1.74 | -4.81 | -0.55 | 0.28 | -1.36 | -3.5 | -1.4 | -5.56 | -1.39 | -0.22 | -2.54 |
|  | 0~2 | -3.4 | 0.24 | -6.91 | -0.69 | 0.16 | -1.54 | -4.45 | -2.19 | -6.66 | -1.94 | -0.68 | -3.18 |
|  | 0~3 | -4.12 | -0.15 | -7.92 | -0.88 | -0.01 | -1.75 | -5.1 | -2.65 | -7.48 | -2.14 | -0.78 | -3.49 |
|  | 0~4 | -3.82 | 0.49 | -7.95 | -0.67 | 0.24 | -1.57 | -4.67 | -2.01 | -7.26 | -1.86 | -0.4 | -3.31 |
|  | 0~5 | -4.12 | 0.53 | -8.56 | -0.47 | 0.49 | -1.42 | -5 | -2.15 | -7.77 | -1.91 | -0.35 | -3.45 |
| upper respiratory | 0 | 1.42 | 4.87 | -1.91 | -0.15 | 0.71 | -1.01 | -1.45 | 0.84 | -3.69 | -0.6 | 0.62 | -1.8 |
|  | 1 | -3.55 | -0.33 | -6.67 | -0.22 | 0.55 | -0.99 | -3.09 | -0.97 | -5.16 | -1.39 | -0.25 | -2.52 |
|  | 2 | -2.75 | 0.28 | -5.69 | -0.28 | 0.42 | -0.97 | -2.36 | -0.31 | -4.37 | -1.04 | 0.03 | -2.09 |
|  | 3 | -2 | 1.02 | -4.92 | -0.33 | 0.34 | -1 | -2.41 | -0.38 | -4.4 | -0.63 | 0.43 | -1.68 |
|  | 4 | -1.1 | 1.89 | -4.01 | 0.07 | 0.75 | -0.61 | -1.86 | 0.16 | -3.85 | -0.62 | 0.43 | -1.66 |
|  | 5 | 0.48 | 3.53 | -2.48 | 0.24 | 0.92 | -0.43 | -0.43 | 1.61 | -2.44 | -0.26 | 0.79 | -1.3 |
|  | 0~1 | -1.58 | 2.34 | -5.36 | -0.25 | 0.73 | -1.23 | -2.9 | -0.41 | -5.33 | -1.37 | 0.01 | -2.73 |
|  | 0~2 | -3.2 | 1.13 | -7.35 | -0.4 | 0.62 | -1.41 | -3.73 | -1.03 | -6.35 | -1.8 | -0.31 | -3.26 |
|  | 0~3 | -4.01 | 0.71 | -8.51 | -0.5 | 0.55 | -1.54 | -4.47 | -1.55 | -7.31 | -1.88 | -0.27 | -3.47 |
|  | 0~4 | -4.04 | 1.11 | -8.92 | -0.33 | 0.76 | -1.41 | -4.5 | -1.32 | -7.58 | -1.77 | -0.03 | -3.48 |
|  | 0~5 | -3.97 | 1.6 | -9.24 | -0.24 | 0.91 | -1.37 | -5.12 | -1.72 | -8.4 | -2.07 | -0.22 | -3.88 |
| lower respiratory | 0 | 3.37 | 8.59 | -1.59 | 0.21 | 1.46 | -1.03 | 1.96 | 5.43 | -1.39 | 2.05 | 3.86 | 0.27 |
|  | 1 | -0.07 | 4.9 | -4.8 | -0.12 | 1.01 | -1.23 | -1.59 | 1.6 | -4.68 | 0.5 | 2.19 | -1.16 |
|  | 2 | -2.9 | 1.55 | -7.16 | -0.18 | 0.83 | -1.17 | -2.55 | 0.47 | -5.47 | -0.94 | 0.63 | -2.49 |
|  | 3 | -0.41 | 4.12 | -4.74 | -0.11 | 0.86 | -1.08 | -0.42 | 2.66 | -3.4 | -0.37 | 1.2 | -1.91 |
|  | 4 | 2.6 | 7.18 | -1.79 | 1.02 | 2.02 | 0.03 | 2.17 | 5.24 | -0.82 | 1.13 | 2.7 | -0.42 |
|  | 5 | 0.75 | 5.32 | -3.62 | 1.1 | 2.11 | 0.11 | 1.46 | 4.55 | -1.55 | 1.3 | 2.88 | -0.25 |
|  | 0~1 | 2.23 | 8.28 | -3.48 | 0.05 | 1.48 | -1.36 | 0.13 | 3.93 | -3.53 | 1.72 | 3.79 | -0.31 |
|  | 0~2 | -0.2 | 6.37 | -6.36 | -0.09 | 1.38 | -1.53 | -1.39 | 2.68 | -5.3 | 0.73 | 2.97 | -1.46 |
|  | 0~3 | -0.42 | 6.76 | -7.11 | -0.13 | 1.37 | -1.62 | -1.45 | 3.01 | -5.7 | 0.43 | 2.85 | -1.94 |
|  | 0~4 | 1.63 | 9.6 | -5.75 | 0.46 | 2.03 | -1.09 | 0.51 | 5.41 | -4.17 | 1.39 | 4.01 | -1.18 |
|  | 0~5 | 1.79 | 10.43 | -6.18 | 0.89 | 2.56 | -0.74 | 0.75 | 6.07 | -4.3 | 1.8 | 4.62 | -0.95 |
| circulatory | 0 | 2.04 | 5.45 | -1.25 | -0.27 | 0.53 | -1.06 | -1.46 | 0.78 | -3.65 | -0.19 | 1.01 | -1.38 |
|  | 1 | -2.61 | 0.54 | -5.67 | -0.45 | 0.27 | -1.16 | -4.01 | -1.97 | -6 | -1.54 | -0.44 | -2.63 |
|  | 2 | -2.26 | 0.63 | -5.07 | -0.68 | -0.04 | -1.32 | -2.78 | -0.83 | -4.69 | -1.39 | -0.37 | -2.4 |
|  | 3 | -0.55 | 2.39 | -3.4 | -0.84 | -0.23 | -1.46 | -2.03 | -0.05 | -3.97 | -1.12 | -0.1 | -2.14 |
|  | 4 | 0.96 | 3.91 | -1.91 | 0.12 | 0.76 | -0.51 | 0.28 | 2.26 | -1.66 | 0.05 | 1.07 | -0.95 |
|  | 5 | -0.28 | 2.66 | -3.14 | 0.34 | 0.98 | -0.3 | 0.04 | 2.02 | -1.91 | 0.2 | 1.23 | -0.81 |
|  | 0~1 | -0.55 | 3.3 | -4.25 | -0.53 | 0.37 | -1.43 | -3.69 | -1.27 | -6.04 | -1.28 | 0.07 | -2.62 |
|  | 0~2 | -1.97 | 2.19 | -5.95 | -0.86 | 0.07 | -1.78 | -4.44 | -1.87 | -6.95 | -1.9 | -0.46 | -3.32 |
|  | 0~3 | -2.05 | 2.47 | -6.38 | -1.17 | -0.22 | -2.11 | -4.93 | -2.15 | -7.63 | -2.28 | -0.74 | -3.8 |
|  | 0~4 | -1.05 | 3.91 | -5.77 | -0.92 | 0.07 | -1.9 | -3.95 | -0.92 | -6.88 | -1.82 | -0.16 | -3.45 |
|  | 0~5 | -1.16 | 4.19 | -6.24 | -0.7 | 0.35 | -1.74 | -3.77 | -0.51 | -6.93 | -1.61 | 0.16 | -3.35 |

| **Table S5** ERs of the single air pollutant models (exceeding 100 µg/m3 of O_3_-8h) | | | | |
| --- | --- | --- | --- | --- |
|  | lag(day) | ERs(%) | | |
|  |  | O_3_ | | |
|  |  | fit | high | low |
| respiratory | 0 | -0.24 | 1.59 | -2.03 |
|  | 1 | -0.46 | 1.25 | -2.15 |
|  | 2 | 0.86 | 2.52 | -0.77 |
|  | 3 | 1.02 | 2.69 | -0.63 |
|  | 4 | 0.17 | 1.83 | -1.47 |
|  | 5 | 2.47 | 4.11 | 0.85 |
|  | 6 | 1.05 | 2.72 | -0.58 |
|  | 7 | 1.41 | 3.08 | -0.23 |
| upper respiratory | 0 | 0.59 | 2.5 | -1.28 |
|  | 1 | 0.63 | 2.41 | -1.12 |
|  | 2 | 1.03 | 2.75 | -0.67 |
|  | 3 | 0.82 | 2.57 | -0.9 |
|  | 4 | 0.75 | 2.49 | -0.97 |
|  | 5 | 3.06 | 4.77 | 1.38 |
|  | 6 | 1.26 | 3 | -0.45 |
|  | 7 | 1.66 | 3.4 | -0.06 |
| lower respiratory | 0 | -0.54 | 2.03 | -3.05 |
|  | 1 | -1.97 | 0.44 | -4.32 |
|  | 2 | 0.51 | 2.86 | -1.78 |
|  | 3 | 0.87 | 3.23 | -1.44 |
|  | 4 | -0.63 | 1.72 | -2.93 |
|  | 5 | 1.76 | 4.11 | -0.54 |
|  | 6 | 0.85 | 3.22 | -1.47 |
|  | 7 | 0.9 | 3.26 | -1.41 |
| circulatory | 0 | -0.8 | 1.67 | -3.21 |
|  | 1 | -1.9 | 0.42 | -4.16 |
|  | 2 | 0.8 | 3.04 | -1.39 |
|  | 3 | 1.83 | 4.08 | -0.37 |
|  | 4 | -0.6 | 1.65 | -2.8 |
|  | 5 | 2.83 | 5.06 | 0.65 |
|  | 6 | 1.82 | 4.06 | -0.37 |
|  | 7 | 1.34 | 3.58 | -0.85 |

**Fig. S1**
